# A model for colour preference behaviour of spring migrant aphids

**DOI:** 10.1098/rstb.2021.0283

**Published:** 2022-10-24

**Authors:** Thomas F. Döring, Sascha M. Kirchner

**Affiliations:** ^1^ Agroecology and Organic Farming Group, University of Bonn, Auf dem Hügel 6, 53121 Bonn, Germany; ^2^ Faculty of Organic Agricultural Sciences, University of Kassel, Nordbahnhofstraße 1a, 37123 Witzenhausen, Germany

**Keywords:** *Brevicoryne brassicae*, colour vision, host, *Myzus persicae*, PLSR, wavelength

## Abstract

Aphids are economically and ecologically important herbivorous insects. A critical step in their life cycle is the visually guided host finding behaviour. To elucidate the role of colour in host finding of aphid spring migrants we conducted large colour trap experiments in the field and analysed aphid catch data, using trap spectral reflectance data as input. Based on known and putative photoreceptor sensitivities we developed and optimized a simple empirical colour choice model for spring migrants of different aphid species which confirmed and explained the yellow preference of these insects. In a further step, we applied multivariate statistical methods to behavioural and reflectance data, but without data on photoreceptor sensitivities, to find the wavelengths of greatest importance for the aphids' behavioural responses. This analysis confirmed the position of the green photoreceptor peak previously obtained independently with electrophysiological methods. In a final step, we applied the colour preference model to a dataset of leaf spectra. This showed that aphid visual preference would be dependent on the plants’ nutritional status, with lower nitrogen input being associated with stronger preference, despite known benefits of high nitrogen levels for aphid reproduction and fitness. Ecological and evolutionary implications of these results are discussed.

This article is part of the theme issue ‘Understanding colour vision: molecular, physiological, neuronal and behavioural studies in arthropods’.

## 1. Introduction

Many aphid species are important pest insects, and after decades of research targeted at improving aphid control, plant damage caused by aphids still remains a challenge in various agricultural and horticultural cropping systems [1]. Aphids also serve as model species for the investigation of fundamental ecological questions, e.g. in the areas of endosymbiosis [2], multitrophic interactions [3], population dynamics [4], or climate change biology [5]. Aphid life cycles are highly diverse, with some species alternating between different host plant species, and many alternating between periods of sexual and asexual reproduction [6]. Aphids are also characterized by sophisticated polymorphism systems, e.g. with wingless individuals specialized in maximizing reproduction, and winged morphs contributing to dispersal [7].

When winged aphids are searching for new host plants, colours play an important role [8]. This was mainly concluded from field experiments in which landing aphids reliably responded to varied artificial colours [9,10]. With a series of field experiments Moericke [9] demonstrated that pure yellow without reflectance in the ultraviolet (UV) attracted the highest number of winged aphids and that fewer aphids responded to orange, yellow-green and green; still lower landing rates were observed on red, blue, purple, white, grey and black. The preference of aphids for the green-yellow wavelength band (around 550 nm) was later confirmed regarding target approaches by walking [11] and flying aphids in the laboratory [12].

Despite the large number of studies on aphid responses to colours, the mechanisms underlying these responses have only in the last two decades begun to be explored. Without understanding the mechanisms, however, it is difficult to predict the response of aphids to a given colour unless exactly this colour is tested for the aphids' behavioural response. This is so because human and insect colour vision are fundamentally different, and colour classification by humans (e.g. into colour categories like ‘yellow’ or ‘green’) is unlikely to coincide with insect colour perception [13].

The first study reporting behavioural responses of aphids to colours [14] provides some of the deepest insights into the underlying mechanisms. In an ingenious experiment, Moericke [14] showed that green peach aphids (*Myzus persicae*), when walking on grey paper, did not respond with probing behaviour, i.e. extending their proboscides and trying to insert their stylets into the paper; however, when walking from a blue paper onto a grey one, grey did elicit the probing response, thereby proving that the aphids' behaviour followed a successive contrast effect. This, in turn, provides indirect but strong evidence that the response of this aphid species to colours is linked to a colour opponent mechanism (COM) [10,13]. Such a COM would be represented by neurons that are excited by the input from one type of photoreceptor (e.g. a green receptor), and are inhibited by the input from another photoreceptor type (e.g. a blue receptor).

Electrophysiological experiments investigated the spectral sensitivity of *M. persicae* [15] and the cabbage aphid *Brevicoryne brassicae* [16], revealing the wavelength position of the maximal sensitivity of the aphids' green receptors (around 530 nm), and the existence of two further receptors, a blue and a UV receptor. Intracellular recordings from the green receptor of the pea aphid *Acyrthosiphon pisum* showed the green receptor to peak at 518 nm [17]. Combining this physiological information and knowledge on COMs promised to be a powerful tool for analysing the behavioural response of aphids to coloured stimuli. We, therefore, aimed to repeat the field experiments pioneered by Moericke, now using modern colour measurement techniques, more systematic variation of colours, and new analytical tools. In a previous study, we had set out water traps painted in 70 different colours in a large-scale field experiment during the autumn and showed that a COM pitting the input of the green against the blue photoreceptor can be used to predict landing responses of aphid autumn migrants to colours [18]. In that experiment we further used reflectance spectra of a large number of plant species to show that, despite lacking a red receptor, aphids would respond differently to colours called ‘red’ and ‘green’ by humans. While this experiment focused on questions of colour preference of aphids that migrate to their winter hosts, and on the preference for green versus red leaves of autumnal trees, we were also curious to know more about colour choice in spring migrants, particularly because the host finding behaviour of these morphs is of much more direct importance in many agricultural crops than that of the autumn migrants.

In particular, we asked: (i) can the landing response of aphids to colour serve to provide *evidence* for a colour opponent mechanism in these insects, especially for species other than *M. persicae*? (ii) would a colour-choice model based on the spring migrant catch generally differ from the one derived from the autumn trapping experiment? (iii) do aphid species differ in their behavioural response to colours, as suggested by previous experiments [19]? Further, we wanted to know (iv) whether aphid and reflectance data alone, i.e. without making assumptions about photoreceptor sensitivities, would be sufficient to reveal any mechanisms underlying the aphids' landing behaviour. Finally (v), we asked what consequences the colour choice behaviour would have for aphid host selection, in particular given that plant leaf colour is associated with the plant's nitrogen status.

To answer these questions, we used a dataset from spring 2007—a field experiment similar to the one conducted in autumn 2007 [18]—and a similar trial from spring 2008, for which data from the pollen beetle *Meligethes aeneus* had already been analysed [20]. We exposed a large number of coloured traps to flight activity of aphids in the field, and used the reflectance spectra of the traps to analyse the trap catch, with or without using data on spectral sensitivities of photoreceptors. Using the resultant colour choice model, we then predicted aphid responses to wheat leaves sampled from different plots with contrasting nutrient supply.

## 2. Material and methods

### (a) Insect trapping

Two insect trapping experiments were performed in the field during aphid spring migration, on days without precipitation. Experiment 1 was conducted over three trapping periods in May 2007 (5–10, 15–18, and 22–24 May) at Silwood Park near Ascot, Berkshire, UK (51.4151 N, −0.6536 E, 66 m a.s.l.) with 140 traps painted in 70 different colours. Experiment 2 was set up in May 2008, over three trapping periods (7–9 May, 12–14 May and 19–21 May) at Rothamsted, Harpenden, UK (51.8053 N, −0.3666 E, 130 m a.s.l.), with 100 traps painted in 50 different colours; of the latter experiment, only data from 98 traps could be included in the analysis. In both experiments, Petri dishes (14 cm diameter) were used as trapping devices; these were fixed to a wooden stand and were placed *ca* 30 cm above the bare soil. Further details on trap design are given elsewhere [18].

The colours were various mixtures of base water-based masonry paints, in particular, yellow, blue, white and black in experiment 1, and additional green and red paints in experiment 2, thereby obtaining several colour series ranging in hue (from red to yellow to green to blue), as well as in saturation and brightness (by mixing white or black to the pure hues). In experiment 2, additional colours with high UV reflectance were created by mixing barium sulfate powder with yellow and green masonry paint and a binder.

The traps were set out in the field in a 2 m × 2 m grid, resulting in four rows of 35 traps and four rows of 25 traps, in experiments 1 and 2, respectively. The experimental layout was a completely randomized design in experiment 1 and a randomized complete block design in experiment 2. The traps were filled with water and an odourless detergent (Lipsol from Bibby Sterilin Ltd., UK) to reduce surface tension.

All winged aphids from 2007 were collected from the traps, stored in 70% ethanol and identified to species level whenever possible, using appropriate taxonomic identification keys [21–23]. Aphids collected in the 2008 experiment were not further identified.

### (b) Plant sampling

Leaves of winter wheat plants (cultivar Primus, sown on 2 October 2013; 300 seeds m^−2^) were sampled for spectral characterization on 6 May 2014 from the International Organic Nitrogen Fertilization Long-Term Trial at Humboldt University Berlin (52.4656 N, 13.2977 E, 51 m a.s.l). The trial, following its German name, is abbreviated as IOSDV [24] and was established in 1984. This long-term static field trial is based on a three-course rotation with potatoes, winter wheat (electronic supplementary material, figure S1) and spring barley, with all three crops grown each year. The soil type is an albic luvisol formed from silty sand above loamy sand, with the pre-trial top soil properties being characterized by low organic carbon content (0.656% C_org_), and a strongly acidic pH (5.4) [24]. The climate is intermediate between oceanic and continental, with annual averages (1971–2000) of 9.6°C air temperature and 540 mm precipitation. In an incomplete factorial design, the trial investigates the interactive effects of organic and mineral fertilization. Factor A (organic fertilization) comprises three levels (without organic fertilization; application of farmyard manure; and crop residue application, i.e. straw and green manuring). Factor B (mineral N fertilization) comprises four levels. Winter wheat receives 0, 60, 110 and 160 kg N ha^−1^ at the four different levels, respectively. Factor levels are combined to obtain 10 fertilization treatments (electronic supplementary material, table S1). With three field replications, there are 30 plots per crop species each year. From each of the 30 winter wheat plots (size: 30 m^2^), six plants were randomly selected and a single leaf lamina was cut from the plant and stored in paper bag until measurement within approximately 2 h.

### (c) Spectral measurements

Reflectance spectra of the water-filled traps of experiments 1 and 2 and the respective background soils were measured with a RAMSES-ARC spectrophotometer (from TriOS GmbH, Oldenburg, Germany) in the range of 320–950 nm in 5 nm steps against a BaSO_4_ white standard. Reflectance spectra of 70 of the 140 traps of experiment 1 are displayed in the electronic supplementary material, figure S2a-d; selected reflectance spectra from experiment 2 have been published previously [20]. Data of all reflectance spectra are given in the electronic supplementary material, table S2.

Reflectance spectra of the upper sides of wheat leaves from the long-term field experiment were measured against a spectralon white standard with an AvaSpec spectrometer (ULS2048X-USB2-UA50 from Avantes, Apeldoorn, the Netherlands, bandwidth: 200–1150 nm, 2.4 nm spectral resolution) connected to a Xenon pulse lamp (AvaLight-XE). Reflectance spectra were determined on six leaves per plot with one measurement per leaf; the spectrometer measured the diffuse reflectance of the sample at an angle of 45° on an area of *ca* 1.7 cm^2^. Reflectance data from the AvaSpec was smoothed using a moving average of 2.4 nm width. Subsequent calculations were performed with data in 5 nm steps between 300 and 700 nm.

### (d) Colour choice modelling and statistical calculations

To build the colour-choice model we first calculated the response variable *y* as the number of aphids *n_t_* in a trap *t* relative to the average number of aphids *n*_ref_ that had been caught per trap in two identical yellow reference traps, *y* = *n*_t_/*n*_ref_. (Trap codes of reference traps were ‘00a’ and ‘00a’ in experiment 1, and ‘Y01A’ and ‘Y01B’ in experiment 2; electronic supplementary material, table S2.) To find the best explanatory variables we converted the colour measurements (reflectance spectra) of the traps into photon catch values for each trap. We calculated the photon catch *P* that a trap *t* elicits in a photoreceptor *R* as

$$P_{R}(t)=\frac{\int I_{t}(\lambda )\;S_{R}(\lambda )\;D(\lambda )\;d\lambda }{\int I_{b}(\lambda )\;S_{R}(\lambda )\;D(\lambda )\;d\lambda },$$

where *I*_*t*_(*λ*) is the reflectance spectrum of the trap *t*; *S*_*R*_(*λ*) the sensitivity function of the photoreceptor *R*, with the sensitivity peak of *R* varying between 320 nm and 610 nm in 10 nm steps; *D*(*λ*) the illumination spectrum (standard sunlight D65); and *I*_*b*_(*λ*) the reflectance spectrum of the background *b* against which the trap stimulus appears [25], in this case, bare soil from the experimental area. Photoreceptor sensitivity curves *S*_*R*_(*λ*) were generated using model templates [26], with a fixed ratio between the half-max bandwidth Δ*_λ_* and the peak wavelength *λ*_max_ (Δ*_λ_*/*λ*_max_ = 0.18), and with no beta peak [18].

Further, using the datasets from the two years, the relationships between the spectral reflectance of different coloured water traps and the number of trapped aphids were investigated using a multivariate approach, without any data on spectral sensitivity entering the statistical models. Relative reflectance from 310 nm to 700 nm in 5 nm steps of the coloured water traps were used as explanatory variables (*X*). The total number of the trapped aphids in each of the coloured water traps was used as response variable (*Y*). The two years were analysed separately. For this analysis, we selected partial least square regression (PLSR) [27] as a reliable technique handling strongly collinear datasets [28]. The optimum number of components was found by visually inspecting plots of root mean squared error of prediction against the number of components in the model.

The program R, v. 4.0.3 was used for all statistical calculations [29]. Nonlinear modelling (i.e. for Gompertz functions) was performed with the *nls* function. PLSR was conducted with the *pls* package [27]. Data from the IOSDV experiment were subjected to ANOVA with subsequent comparison of means with Tukey's HSD test.

## 3. Results

### (a) Aphid catch: species composition

In experiment 1, a total of 17 383 winged aphid individuals from 98 aphid taxa was found. The catch was dominated by *B. brassicae* (40.4%) and *M. persicae* (23.9%). A minority of individuals (12.8%) could only be identified to genus level (mainly *Aphis* sp. and *Brachycaudus* sp.), and a further 207 individuals remained unidentified. The majority of taxa (63 out of 98) and individuals (90.8%) belonged to the tribus Macrosiphini. Seven species, contributing 30.6% of individuals to the trap catch, were very polyphagous species (feeding on more than three plant families), whereas 26 species (2.3% of individuals) were strictly monophagous (feeding on one plant species only). The maximal catch per trap (summed over the three trapping periods) was 527 individuals in a yellow trap. In 2008, a total of 1732 aphids were caught; here, the maximal catch per trap over the three trapping periods was 79 individuals in a yellow trap.

### (b) Landing behaviour follows a colour opponent mechanism

**Figure 1.**
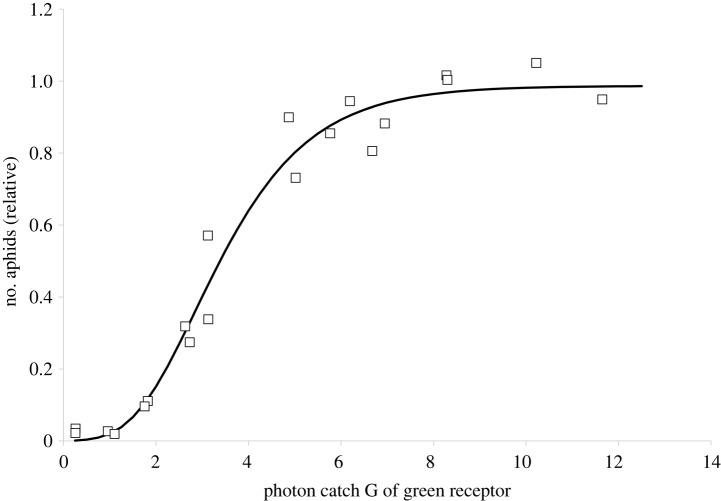
For 20 traps painted in mixtures of yellow and black (reflectance spectra see the electronic supplementary material, figure S2), the photon catch of a green receptor (with maximal sensitivity at 530 nm) was calculated (*x*-axis) and plotted against the aphid catch (relative to the maximum catch). The model follows a Gompertz function (equation see text).

To test whether the aphid landing behaviour in the trap experiment follows a COM, we performed the data analysis in the following two steps:

(i) when yellow paint is mixed with varied amounts of black paint (i.e. colours ranging from pure yellow to pure black), the reflectance is very low in the UV and blue (electronic supplementary material figure S2). This means that traps painted in the colours of this ‘yellow-to-black’ series will almost exclusively excite the green receptor, whereas the two other receptors (with sensitivity peaks in the UV and blue) will not be excited. Available electrophysiological data [15,16] shows that *M. persicae* and *B. brassicae*, i.e. the two dominant species in the trap catch, possess a green receptor with a peak sensitivity around 530 nm. If the landing behaviour is linked to the green receptor input, we expect a correlation between aphid numbers and photon catch of the 530 nm green receptor. Importantly, we would expect this to be the case both *with or without* a COM, because whether or not the input from the blue or UV receptor feeds into a COM, in both cases the contribution will be negligibly small. What we found was indeed a strong correlation between aphid catch and the photon catch of a green photoreceptor with peak sensitivity at 530 nm, for this subset of traps. When all aphid species were pooled, the relationship followed a sigmoidal curve and could be modelled with a Gompertz function (*y* = 0.987 exp(−8.12 exp(−0.733 G)), where G is the photon catch of the green receptor (residual standard error 0.069; d.f. = 17, *p* < 0.0001) (figure 1). Table 1 (upper half) gives the respective functions for all aphid taxa with a total catch of more than 500 individuals; and
(ii) in a second step we applied the Gompertz model of step (i), derived from the yellow-to black subset of traps, to *all* traps, i.e. to predict the aphid catch that the input from the green receptor alone would generate. If the aphids' behaviour followed the input from their green receptor *only*, we would expect that for all traps the resulting model would be more or less the same as in the yellow-to-black series subset; the residuals of the regression function would be small, i.e. the differences *e_t_* between ${\hat{\;y}}_{G}$, the number of aphids predicted from the yellow-to-black series model, and *y_t_* the actually observed number of aphids (*e_t_* = *y_t_* – ${\hat{y}}_{G}$). Particularly, these residuals *e_t_* would *not correlate* with any further spectral information of the traps, i.e. with the input from any other photoreceptor.

**Table 1.** Statistical parameters for the Gompertz models exemplified in figure 1 (upper half of the table), and for the correlation as shown in figure 2. (Above: d.f. = 17, below: d.f. = 138.)

	aphid taxon	*all*	*Aphis*	*Brachycaudus*	*Brevicoryne*	*Cavariella*	*Hyperomyzus*	*Macrosiphum*	*Myzus*
		*aphids*	sp*.*	sp*.*	*brassicae*	*aegopodii*	*lactucae*	*euphorbiae*	*persicae*
	*n*	17 383	571	1252	7018	558	512	762	4157
Gompertz model for yellow-to-black trap series	a: estimate	0.987	1.211	0.878	1.075	0.921	1.159	1.125	1.001
	a: std error	0.035	0.123	0.141	0.047	0.105	0.154	0.106	0.051
	b: estimate	−8.116	−8.939	−2.809	−15.893	−6.730	−8.318	−9.011	−7.208
	b: std error	2.471	11.047	0.997	12.493	3.219	5.728	5.219	1.832
	c: estimate	−0.733	−0.907	−0.395	−1.054	−0.493	−0.545	−0.606	−0.544
	c: std error	0.111	0.495	0.165	0.292	0.142	0.197	0.175	0.079
	residual std error	0.069	0.305	0.169	0.122	0.129	0.207	0.162	0.071
linear model of residuals against input from blue receptor	intercept: estimate	0.060	0.130	0.078	0.073	0.064	0.130	0.052	0.072
	intercept: std error	0.020	0.048	0.029	0.027	0.020	0.032	0.025	0.019
	slope: estimate	−0.074	−0.080	−0.044	−0.091	−0.068	−0.081	−0.073	−0.071
	slope: std error	0.004	0.009	0.005	0.005	0.004	0.006	0.005	0.003
	*p*-value	2 × 10^−16^	1.56 × 10^−15^	2.51 × 10^−13^	2 × 10^−16^	2 × 10^−16^	2 × 10^−16^	2 × 10^−16^	2 × 10^−16^
	adjusted R squared	0.742	0.366	0.318	0.700	0.715	0.574	0.642	0.753
	residual std error	0.158	0.381	0.233	0.216	0.156	0.253	0.196	0.147

**Figure 2.**
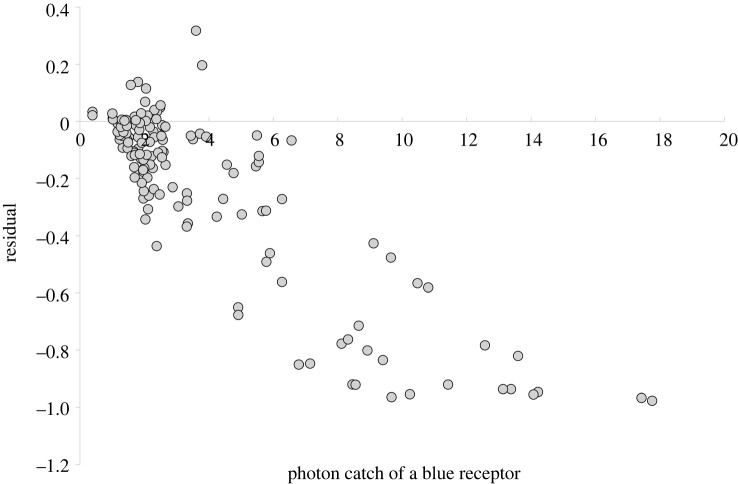
Residuals *e_t_* as indicated in figure 1, and plotted against the photon catch of a blue receptor (*λ*_max_ = 450 nm).

If, on the other hand, a COM was responsible for the aphids’ landing behaviour, we would expect the residuals *e_t_* to be *negatively correlated* with the input from a blue or UV receptor (or both). As can be seen in figure 2, the residuals showed a significant negative correlation with the input from a blue receptor (with a peak sensitivity chosen at 450 nm), thereby providing evidence for a COM driving the aphids' behaviour. When all species are considered together, the correlation coefficient for a linear model of this relationship is *r* = –0.862 (*p* < 0.001, d.f. = 138). Although the relationship is curvilinear, we chose a linear model for the sake of simplicity; alternative curvilinear models do not result in a different sign of the relationship. For all seven species with more than 500 individuals the relationship between the residuals *e_t_* and the blue receptor input B is significantly negative (table 1, bottom half).

**Figure 3.**
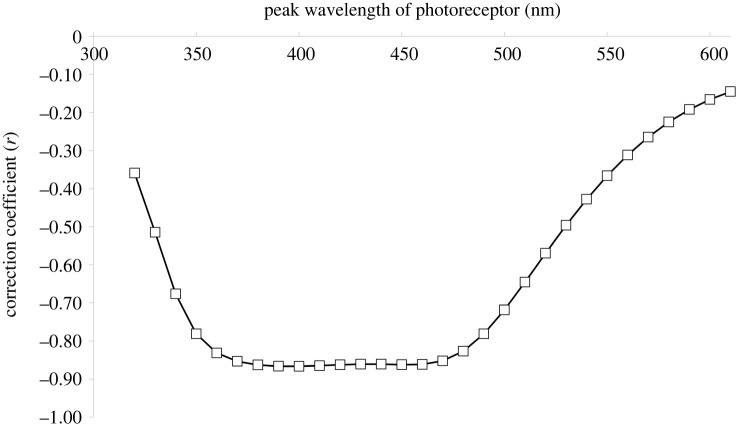
Correlation between residuals *e_t_* (as indicated in figure 1) and the photon catch of several photoreceptors, plotted against the respective wavelength of the peak sensitivity *λ*_max_ of these photoreceptors. The example of a negative correlation displayed in figure 2 for one blue receptor is generalized for all receptors in this graph.

Currently, the position (peak sensitivity) of the blue receptor in aphids is unknown, as intracellular recordings have not been performed in aphid blue receptors. Thus, the correlation exemplified in figure 2 and table 1 between the residuals (of the model built with the green receptor input alone) and the input from the blue photoreceptor is only one possible case. Therefore, the respective correlation was calculated for *all* photoreceptors, i.e. for receptors with their peak sensitivity ranging between 320 and 610 nm (in 10 nm steps). When the correlation coefficient is displayed against the peak sensitivity of the receptor, the resulting correlogramm shows that the negative relationship between residuals and photon catch is most marked with input from photoreceptors that peak between 380 and 470 nm, i.e. mainly in the blue, (figure 3), whereas the correlation is weaker with input from photoreceptors that have peaks at larger (greater than 480 nm) or smaller (less than 370 nm) wavelengths.

### (c) Model development and comparison of alternative models

Having established that the behaviour of at least seven aphid taxa followed a COM with positive input from a green receptor and negative input from a blue or UV receptor, we looked at various mathematical representations of COMs as explanatory variables and tested several alternative models. Given the sigmoidal response in both figures 1 and 2, a COM representation that uses the difference of two Gompertz functions would be straightforward. However, this would require as many as six parameters to model the insects’ behaviour. Striving for a more parsimonious model, we therefore looked at simpler models with fewer parameters.

**Figure 4.**
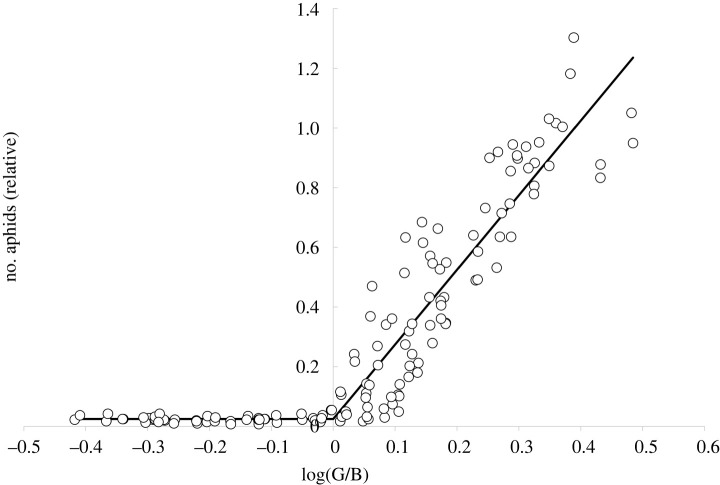
Number of aphids relative to the reference catch plotted against the expression log(G/B) where G is the photon catch of a green receptor (*λ*_max_ = 530 nm) and B is the photon catch of a blue receptor (*λ*_max_ = 470 nm). Equation of graph see text.

One possible representation of a COM is the expression log(G) – log(B) = log(G/B), i.e. the log ratio between the photon catch G of the green photoreceptor and the photon catch B of the blue photoreceptor [20]. When the number of aphids relative to the reference catch is displayed against the respective value of log(G/B) of each trap, the relationship between the two variables can be modelled with a simple piecewise regression, with a split point at log(G/B) = 0, i.e. where G = B (figure 4). Here, the best fit was found for *λ*_max_(B) = 470 nm, whereas *λ*_max_(G) was held fixed at 530 nm again (electronic supplementary material table S3). For the left-hand part of the model (G < B), the slope was found to be not significantly different from 0. Thus, with *y* being the number of aphids relative to the reference trap, the model had the shape *y* = *a* log(G/B) + *b*, for G > B, and *y* = *b* else, with *a* = 2.499 ± 0.071 and *b* = 0.025 ± 0.001 (mean ± s.e., *r*^2^ = 0.890, n = 140).

Finally, we tested whether residuals *u_t_* between the values ${\hat{y}}_{M}$ predicted by the split linear regression model M and the observed values *y_t_* of the aphid catch (*u_t_* = *y_t_* – ${\hat{y}}_{M}$) were still correlated with the photon catch of any modelled photoreceptors R (peak sensitivities at *λ*_max_ = R). This was not the case. The maximal correlation was *r* = 0.159 at 500 nm (*p* = 0.06, not significant), and the minimal correlation was *r* = –0.076 (*p* = 0.37, not significant) at 320 nm. There was also no significant correlation between the summed input from all three photoreceptors together (UV (350 nm), blue (470 nm) and green (530 nm)) and these residuals *u_t_* (*r* = 0.135, *p* = 0.112, not significant). Therefore, we conclude that the model is complete in terms of spectral input information and the split regression model is sufficient to explain the aphids' response to the coloured traps.

### (d) Model validation with experiment 2

We first tested if the data from experiment 2 followed the same general function as the 2007 data. As the electronic supplementary material, figure S3 shows, this was the case. With the same photoreceptor combinations as before (G: *λ*_max_ = 530 nm and B: *λ*_max_ = 470 nm), the 2008 data was modelled with a split linear regression function based on *y* = *a* log(G/B) + *b*, for G > B, and *y* = *b* else, with *a* = 1.602 ± 0.092 and *b* = 0.224 ± 0.015 (mean ± s.e., *r*^2^ = 0.689, *n* = 100). When the model parameters from the 2007 model (figure 4) were used to predict relative aphid numbers from 2008 based on the spectral reflectance of the 2008 traps, the agreement between predicted and observed aphid data was good, with an *r*² of 0.689, though slightly biased, with the regression function between observed (*y*) and predicted (*x*) values following the function *y* = 0.711 *x* + 0.151.

### (e) Partial least square regression

**Figure 5.**
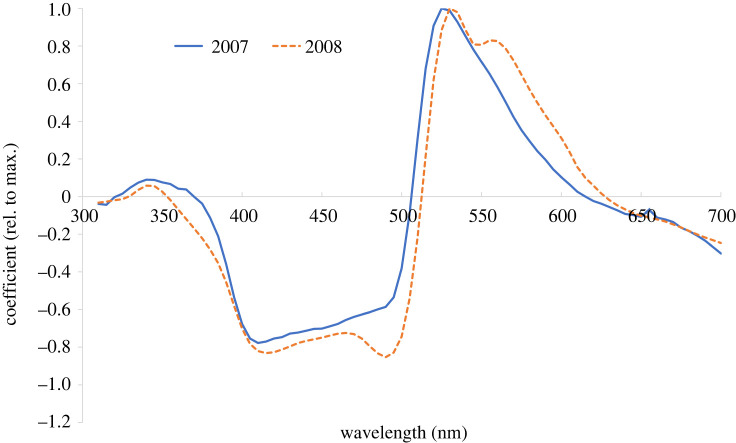
Partial least square regression (PLSR) coefficients for combined aphid catch and spectral reflectance data of the coloured water traps for experiments 1 and 2, plotted against wavelength. (Online version in colour.)

The multivariate regression outputs are presented for models with three components; fewer components led to substantially decreased percentage of explained variation, while a higher number of components resulted in multiple peaks across the spectrum that were inconsistent between the two years. With three components, the *r*^2^ obtained for the PLSR of experiment 1 was 0.730. That is, 73.0% of the total variation in the response variable (*Y*, the aphid counts) was explained by the explanatory variables (*X*, i.e. the reflectance spectra); for experiment 2, this value was slightly lower (63.3%). The contribution of individual wavelengths to the model was assessed using PLSR coefficients; to allow direct comparisons between the two experiments, coefficients were normalized, i.e. for each experiment, individual coefficient values were divided by the respective maximum coefficient of the experiment. Across wavelengths, distinct local maxima of the coefficients were located in the green region, at 525 and 530 nm for experiments 1 and 2, respectively (figure 5). For both experiments, plateaus of negative coefficients were found in the blue region, at 410–490 nm and 415 to 490 nm, for experiments 1 and 2, respectively. Coefficients were small (−0.3 < *x* < 0.3) in the UV (here at wavelengths <380 nm) and in the yellow to red region (at >600 nm).

### (f) Comparison of aphid species

Because the accuracy of the colour choice model is expected to depend on the number of individuals caught, we restricted species comparisons to three groups: *M. persicae*, *B. brassicae* and all other species pooled. In a covariance analysis for the split regression model, no significant differences between species were found in the right-hand slope (i.e. the parameter *a* for the condition G > B), but the intercept was significantly different between species. However, it can be shown that when comparing the attractivity (i.e. the predicted *y*) of any two coloured surfaces with the model, the interspecific differences in the intercepts cancel out.

For the PLSR analysis, the general shape of the coefficient spectra for the taxa listed in table 1 was similar to that shown for all aphids together in figure 5; coefficients were small in the UV, negative in the blue, peaked in the green, and were small greater than 600 nm. Regression coefficients for all taxa shown in table 1 were maximal at 525 nm, and were negative between 380 and 500 nm.

### (g) Application of model to plant leaf spectra

**Figure 6.**
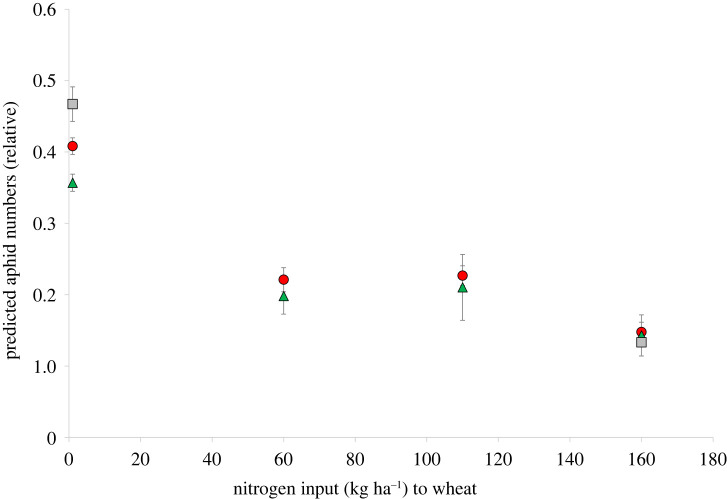
Number of aphids relative to the number in a yellow reference trap (means and standard deviation, *n* = 3 field replicates), as predicted by the model from figure 4 with wheat leaf spectra as input data; wheat leaves were sampled from differently fertilized plots of the IOSDV long-term experiment, with different levels of mineral fertilizer input (*x*-axis), and additional farmyard manure application (red circles), additional green manure and straw application (green triangles), or no organic inputs at all (grey squares). (Online version in colour.)

When the colour choice model developed from experiment 1 was applied to wheat leaf spectra from the fertilization field experiment, the relative number of aphids predicted by the model, based on the leaf spectra as input data, was found to depend on the nitrogen fertilizer level (figure 6). In particular, with the highest nitrogen input level (160 kg ha^−1^), the relative number of aphids was significantly lower than with any of the other nitrogen input levels (Tukey HSD test).

## 4. Discussion

As we have shown, it is not only possible to model the aphids’ response to coloured targets based on a COM (figure 4), but a COM is required to explain this behaviour (figures 2 and 3). Evidence for the COM in aphids has previously been presented for one aphid species, *M. persicae*, based on laboratory studies [13], as well as for autumn migrants, based on trap catches [18]. Here, we have shown that COM is more widespread among aphids and that it explains the behavioural response of aphid spring migrants of at least six more taxa to coloured targets (table 1).

### (a) Comparative interpretation of models

We presented two alternative and complementary approaches of analysing the behavioural response of aphids to coloured targets. The first approach was to build a model using input based on physiologically derived sensitivity data, in particular using knowledge about the peak sensitivity of a green receptor at or near to 530 nm [15–17]. Here, we used the expression log(G/B), a mathematical representation of a COM, as an explanatory variable, i.e. the log ratio of the photon catches of the green and blue receptor. The model follows a piecewise linear regression, with a split point at G = B (figure 4). An alternative way of relating aphid numbers against a COM is a logistic function of the ratio G/B [18]. Which of these mathematical representations is physiologically more meaningful is currently difficult to gauge, in absence of further physiologically based information regarding the mechanisms of the aphids' behaviour towards colours. However, both models have their advantages. While the logistic model may allow a better representation of saturation effects at high G/B-values, the split regression model is mathematically simpler.

The second modelling approach was entirely driven by reflectance data, i.e. did not make use of input from physiological sensitivity functions; thus, this entirely empirical approach was blind to physiological knowledge and may therefore be used to test if the assumptions on physiological sensitivity functions in aphids are reasonable. In fact, the PLSR approach showed a remarkable congruence with the physiological data on peak sensitivities. For both years, the maximum regression coefficient (at 525 and 530 nm, in experiments 1 and 2, respectively, figure 5) was observed very close to or on the same wavelength as the electrophysiologically determined values, namely 530 nm for *M. persicae* [15] and 520–530 nm for *B. brassicae* [16], two aphid species that dominated the 2007 catch (table 1). Further, the PLSR confirms the antagonistic nature of blue reflectance for the response of aphids, since coefficients in this spectral region are negative (figure 5).

### (b) The role of blue versus ultraviolet and the position of the blue receptor

An important question open to debate is the relative importance of UV versus blue reflectance for the responses of aphids to coloured stimuli [18,30]. In particular, it is unclear whether UV or blue or both are contributing to the COM underlying aphid behavioural responses to colours. This is because UV and blue reflectance produced with the previously chosen paints [18] were strongly correlated with each other. With the trap colours chosen for experiment 1, strong correlation between blue and UV reflectance was also observed, and it is therefore not surprising that model optimization did not allow us to differentiate clearly between blue and UV effects, especially at UV reflectance above 350 nm (figure 3). However, colours in experiment 2 were chosen to decrease correlation between UV and blue reflectance by adding a high-UV component to some of the colour mixes. In addition, the PLSR approach helped to tackle this problem of collinearity [28] and revealed, for both experiments, that blue plays a dominant role and UV is less important (figure 5). Previously, it has been argued that it may not be essential to disentangle effects of UV versus blue for aphids, since reflectance in the UV and blue are usually strongly correlated in leaves anyway [30]; however, this correlation is not perfect, especially at higher reflectance values [8]. In any case, to solve this question further experiments will be necessary that use target colours with (almost) uncorrelated variation of blue and UV reflectance.

Because the spectral position of the blue and UV photoreceptors in aphids are not yet confirmed physiologically, we attempted to find the position of a short-wavelength photoreceptor by optimizing the model fit (electronic supplementary material table S3). However, probably because of strong collinearity within the reflectance values across the blue region in both experiments 1 and 2, it was impossible to determine the position of the blue receptor by model optimization (figures 3 and 5).

### (c) Comparison of aphid species

We did not find significant differences between aphid species in their colour behaviour. This is in contrast to previous reports, as differences between aphid species in their colour preference have been demonstrated on several occasions. For instance, *Hyalopterus pruni* was more strongly attracted to yellow when the colour was unsaturated, i.e. when mixed with white lead whereas this was not the case with *Aphis fabae* [19]. According to [31], *Rhopalosiphum padi* was more attracted to green than to yellow, whereas *Sitobion avenae*, *Rhopalosiphum maidis* and *Schizaphis graminum* preferred yellow over green. At present it is difficult to explain the discrepancy between our results and previous findings. However, both cited studies include grass feeding aphid species, which, in our investigation were not numerous enough for rigorous inter-species comparisons. This is of particular relevance since grass-feeding herbivores have already been hypothesized as a group that is distinct in the response to colours [32]. More targeted laboratory studies that do not depend on the aphid fauna landing in traps will be needed to elucidate this question.

Regarding seasonal differences in aphid responses to colours, the comparison of autumn catch [18] and spring catch (this study, experiment 1) on the same site with almost identical methods would have been promising. However, because of the almost completely different species composition of the spring and autumn catch, a direct comparison of the models regarding seasonal effects was not possible. Still, the general model shape observed in the autumn and spring model was similar, both indicating that a colour opponent mechanism drives the aphids’ response to colours.

### (d) Ecological implications: aphids and leaf colours

Application of the aphid colour choice model to wheat leaves sampled from different fertilization treatments revealed that low nitrogen fertilizer levels would result in leaf colours being more attractive to aphids than leaves from higher nitrogen input levels (figure 6). At first sight, this is surprising, since aphid reproductive fitness strongly responds to nitrogen [33–35], and the colour choice behaviour would therefore lead aphids to land on host plants with low suitability for reproduction. However, this result needs to be interpreted with caution, for two reasons. First, wheat is a member of the grass family, and based on the arguments presented above, the associated specialized aphid fauna may respond differently to colours compared to the aphid species dominating the catch in the presented colour trap experiments. Second, we did not sample aphid populations on the wheat in the fertilization experiment and have therefore no direct evidence on aphid performance from this field trial. On the other hand, however, the results obtained here (figure 6) may still be of significance, since (i) low nitrogen levels cause yellowing in many non-cereal crop species as well [36], i.e. spectral reflectance changes are similar to those observed here in wheat; and (ii) the response of aphid population growth to nitrogen is relatively robust. The apparently non-adaptive yellow preference confirmed in our experimental data and in the model has been interpreted as the consequence of yellow being a ‘supernormal’ stimulus [37]; the potential adaptive role of yellow preference, however, still remains open.

In this context it is relevant that we investigated spring migrants rather than autumn migrants, as well as leaf colours in the corresponding season. Previous research on the adaptive role of aphid colour choice has much focused on autumnal leaf colours [38–40]. While yellowing of leaves occurs at senescence, this is not typical during the time of the year we focused on; potential mechanisms proposed to explain yellow preference of aphids during autumn, such as the nitrogen re-translocation during leaf senescence [41] may therefore not be relevant in our case. Without a second long-wavelength receptor (i.e. a red receptor), aphids are not able to prefer green over yellow [18]. While potential constraints for the development of red receptors and additional neural processing have been discussed [18], it is conceivable that other, yet unexplored evolutionary forces may have shaped the mysterious preference for yellow over green.

More generally, our colour-choice model predicts that plant leaves with higher reflectance in the blue domain, leaves with whitish wax layers, or very dark green leaves are less attractive for aphids than leaves with higher reflectance in the green spectral domain (around 550 nm) and a lower proportion of blue reflectance. The model could therefore be used to optimize strategies for aphid control [37]. Further studies will be required to generalize the colour-choice model. In particular, the presented model was developed with a constant background of bare soil, while altered colour contrast is expected to interfere with aphid behaviour as well [42,43]. This could then be used in plant mixtures and dense plant stands with the direct colour effects [43].

## 5. Conclusion

As shown, the landing response of spring migrant aphids to colour provided evidence for a colour opponent mechanism. Further, we demonstrated that the colour choice model based on the spring migrant catch agreed with the one derived from a similar trapping experiment conducted in the autumn, which targeted a different stage in the life cycle of aphids. Among the species found in our experiment, the behavioural response to colours did not significantly differ. Finally, aphid and reflectance data alone, i.e. without information on photoreceptor sensitivities, indirectly confirmed the position of the green photoreceptor and provided further confirmation of the COM driving aphid landing behaviour. This means that for taxa where photoreceptor data are absent, the PLSR approach, together with a set of carefully chosen colour stimuli, may help to reveal mechanisms in response to colours.

## Data Availability

Original data are supplied in the electronic supplementary material [44].
